# Association of high adherence to vegetables and fruits dietary pattern with quality of life among Chinese women with early-stage breast cancer

**DOI:** 10.1007/s11136-021-02985-0

**Published:** 2021-09-16

**Authors:** Yuan-Yuan Lei, Suzanne C. Ho, Carol Kwok, Ashley Cheng, Ka Li Cheung, Roselle Lee, Frankie K. F. Mo, Winnie Yeo

**Affiliations:** 1 Department of Clinical Oncology, Prince of Wales Hospital, The Chinese University of Hong Kong, Shatin, New Territories, Hong Kong SAR China; 2 Division of Epidemiology, The Jockey Club School of Public Health and Primary Care, The Chinese University of Hong Kong, New Territories, Hong Kong SAR China; 3 Department of Clinical Oncology, Princess Margaret Hospital, New Territories, Hong Kong SAR China; 4 State Key Laboratory in Oncology in South China, Faculty of Medicine, Hong Kong Cancer Institute, The Chinese University of Hong Kong, New Territories, Hong Kong SAR China

**Keywords:** Breast cancer, Dietary pattern, Quality of life, Chinese women

## Abstract

**Purpose:**

Dietary intake and patients’ quality of life (QoL) are important supportive care issues in breast cancer survivorship. This study aimed to identify dietary pattern before and after breast cancer diagnosis. In addition, the association between dietary patterns and QoL were cross-sectionally and longitudinally investigated.

**Methods:**

A breast cancer cohort which included 1462 Chinese women were longitudinally interviewed at four time-points, namely baseline, 18-, 36-, and 60 months after diagnosis. At each follow-up, validated food frequency questionnaires (FFQ) were used to assess patients’ dietary intake, and factor analysis was used to derive dietary patterns. European Organization for Research and Treatment of Cancer Quality of Life Questionnaire Core 30 (EORTC QLQ-C30) were used to measure QoL at each follow-up. This study included 1368, 1226, 1079 and 1095 patients with invasive disease who completed assessment at baseline, 18-, 36- and 60-month follow-up and had detailed data of dietary intake and QoL.

**Results:**

Based on data obtained at 18-month follow-up, two major dietary patterns were identified: “grain and animal food pattern” and “vegetables and fruits pattern”. Similar dietary patterns were obtained at baseline, 36- and 60- month follow-up. Generalized Estimating Equations (GEE) were used to analyze the longitudinal associations between dietary patterns and QoL over the four follow-ups. High intake of grain and animal food was inversely associated with scores for role functioning (B = − 0.744; 95%CI − 0.147 to − 0.017), dyspnea (B = − 0.092; 95%CI − 0.092 to − 0.092) and constipation (B = − 1.355; 95%CI − 2.174 to − 0.536). Vegetables and fruits intake were positively associated with scores for global health status/QoL (B = 1.282; 95%CI 0.545–2.019), physical functioning (B = 0.545; 95%CI: 0.037–1.053), emotional functioning (B = 1.426; 95%CI 0.653–2.200) and cognitive functioning (B = 0.822; 95%CI 0.007–1.637), while inversely associated with scores for nausea and vomiting (B = − 0.382; 95%CI − 0.694 to − 0.071), dyspnea (B = − 0.570; 95%CI − 0.570 to − 0.570), insomnia (B = − 1.412; 95%CI − 2.647 to − 0.177), loss of appetite (B = − 0.722; 95%CI − 1.311 to − 0.132), constipation (B = − 2.028; 95%CI − 2.775 to − 1.281) and diarrhea (B = − 0.929; 95%CI − 1.481 to − 0.377).

**Conclusion:**

This study suggested that high adherence to “grain and animal food pattern” or “vegetables and fruits pattern” was significantly associated with several aspects of QoL. For instance, vegetables and fruits pattern appears to have beneficial effect on global health status/QoL among Chinese breast cancer patients. Prospective follow-up data could further confirm whether a specific dietary pattern has impact on cancer outcomes.

**Supplementary Information:**

The online version contains supplementary material available at 10.1007/s11136-021-02985-0.

## Plain English summary

In recent decades, dietary intake and patients’ quality of life (QoL) are important supportive care issues in breast cancer survivorship. This study was based on prospective cohort study, which enrolled 1462 Chinese women with early-stage breast cancer. In the present analysis, it first identified the pre- and post-diagnosis dietary pattern, and cross-sectionally investigated the associations between dietary patterns and QoL at 18-month follow-up, then longitudinally analyze the associations between dietary patterns and QoL over the four follow-ups during the first five years of survival. Two major dietary patterns were identified by factor analysis, termed “grain and animal food pattern” and “vegetables and fruits pattern”. The results indicated that each dietary pattern was associated with several aspects of QoL. Higher intake of grain and animal foods was related to worse role functioning, while less symptoms in dyspnea and constipation. In addition, higher consumptions of vegetables and fruits were associated with better global health status/QoL, physical functioning, emotional functioning and cognitive functioning, as well as less severe symptom in nausea and vomiting, dyspnea, insomnia, loss of appetite, constipation and diarrhea. The present findings hint to a potential beneficial effect on QoL for breast cancer patients following a healthy lifestyle habit.

## Introduction

Hong Kong is a relatively more westernized and urbanized city in China. Breast cancer is the most common cancer and the third leading cause of cancer-related mortality in local females [1]. During the three decades between 1976 and 2010, the age-standardized breast cancer incidence rate have increased on average by 1.69% per year, while the mortality rates have decreased on average by 0.02% per year [2]. Epidemiology study estimated that this trend would continue in the next 15 years. Under such circumstances, the population of breast cancer survivors in Hong Kong would grow substantially [2]. While the improvement of breast cancer survival has been encouraging, a critical new issue is in great need of attention; that is, survivorship of the growing population of breast cancer patients, with dietary intake and QoL both being important aspects to be addressed.

Several cross-sectional studies have investigated the association between specific food item and QoL in breast cancer survivors. A Chinese study reported that higher vegetables and fruits intake were significantly associated with better QoL in breast cancer survivors [3]. Blanchard et al. also assessed the association between fruits and vegetables intake and QoL among cancer survivors in the United States (US) [4]. Patients were defined as meeting the American Cancer Society (ACS) 5-A-Day recommendation if they consumed at least five servings of fruits and vegetables per day. The results showed that breast cancer survivors who met the 5-A-Day recommendation had slightly yet significantly higher QoL compared with those who did not [4].

Given food items are consumed in combination and their complex effects are likely to be interactive or synergistic, the focus of nutrition research has shifted from specific food item or nutrient to the overall diet and diet pattern in recent years [5, 6]. Dietary pattern analysis investigated the effect of the overall diet by integrating a comprehensive food or nutrients intake [5, 6]. Three cohort studies have investigated the association between dietary pattern and breast cancer outcome [7–9]. Two of them were conducted in US women, and similar dietary patterns were identified: “Prudent dietary pattern” (characterized by higher intake of fruits, vegetables, whole grains) and “Western dietary pattern” (characterized by higher intake of refined grains, processed meat, red meat). The results showed that higher adherence to the “Prudent dietary pattern” and lower adherence to the “Western dietary pattern” might protect against mortality from causes unrelated to breast cancer [7, 8]. Another study was undertaken in German post-menopausal women with breast cancer. The “healthy dietary pattern”, namely high consumption of vegetables, fruits, vegetable oil, sauces/condiments, and soups/bouillons, was inversely associated with overall mortality and breast cancer recurrence among patients with stage I–IIIA. On the other hand, higher consumption of “unhealthy dietary pattern” was associated with an increased risk of non-breast cancer mortality [9]. While the effect of dietary pattern on QoL has also been explored, the evidence on potential association has been limited. Only a Korean study has explored such association in women with breast cancer [10]. Up to now, the association between post-diagnosis dietary pattern and breast cancer survival or QoL has not been reported in Chinese women.

Based on a prospective breast cancer cohort, this study aimed to first identify the dietary patterns at baseline, 18-, 36- and 60-month follow-up, then cross-sectionally investigate the associations between dietary patterns and QoL at 18-month follow-up, and finally longitudinally analyze the associations between dietary patterns and QoL over the four follow-ups.

## Methods

### Patients

A prospective cohort study named the Hong Kong NTEC-KWC Breast Cancer Survival Study (HKNKBCSS) was initiated in 2011. This enrolled Chinese women with early-stage breast cancer, and aimed to investigate whether soy isoflavones and other lifestyle factors were related to breast cancer outcomes [11–14]. The eligibility criteria were defined as follows: histologically confirmed breast cancer with American joint Committee on Cancer (AJCC) stage 0–III diagnosed within 1 year before study entry [15], any age, female gender, Chinese ethnicity, able to read Chinese, had Eastern Cooperative Oncology Group (ECOG) performance status (PS) 0–1, without severe gastrointestinal disorders, had no prior history of breast or other cancers.

In total, the study cohort recruited 1462 patients from two regional cancer centers in Hong Kong. Each participant provided written informed consent. Enrolled patients were interviewed at four time-points: baseline at study entry, 18-month, 36-month and 60-month follow-up post-diagnosis. The study was approved by the Joint CUHK-NTEC Clinical Research Ethics Committee and the KWC Research Ethics Committee of the Chinese University of Hong Kong and the Hong Kong Hospital Authority.

Baseline assessment were undertaken within 12-month after diagnosis. A total of 1462 patients completed assessment at baseline. The 18-month follow-up was conducted between 12 and 24 months after breast cancer diagnosis, during which 1310 patients in the study cohort completed assessment at that time-point. Participants who had incompletely filled questionnaire for QoL (*n* = 4), who reported implausible dietary intake (energy intake estimates < 500 or > 4000 kcal per day; *n* = 3) or who had carcinoma in situ (*n* = 77) were excluded, resulting in 1226 patients for the present analysis. The 36- and 60-month follow-ups were conducted between 30–42 months and 54–66 months after breast cancer diagnosis, respectively. Using similar inclusion criteria, 1368, 1079 and 1095 patients were included in the longitudinal analysis at baseline, 36- and 60-month follow-up, respectively.

### Data collection at each assessment

Trained personnel conducted face-to-face interview at baseline and each follow-up. During each interview, socio-demographic data were collected by structured questionnaires, and included age, marital status, occupation, education level, family income, menopausal status and prior medical history. Validated questionnaires were used to collect information about lifestyle factors, such as dietary intake and level of physical activity. At each assessment, body weight and height were also measured. Body mass index (BMI) classification was based on criteria adopted in the Asia–Pacific region, which consisted of four groups, as follow: underweight < 18.5 kg/m^2^, normal 18.5–22.9 kg/m^2^, overweight 23–24.9 kg/m^2^ and obese ≥ 25 kg/m^2^ [16]. Patients’ clinical information on breast cancer was retrieved by reviewing hospital medical records.

### Dietary intake and dietary pattern assessment

Dietary intake was collected by filling a validated food frequency questionnaires (FFQ) during face-to-face interview [17]. At baseline, patients recalled their dietary intake over the proceeding 12 months before breast cancer diagnosis. At 18-, 36- and 60-month follow-up, patients recalled their post-diagnosis dietary intake, namely over the proceeding 12 months before each assessment. If patients had cancer recurrence during 18-, 36- or 60-month follow-up, they would not be asked to complete the FFQ, and not included in the present analysis. The FFQ contained 109 food items that were commonly consumed in Hong Kong population and a specific proper portion size was used for quantification of each food item. Based on the design of FFQ, patients should report the frequency of consumption and average amount of intake at each time for each food item. Interviewers would provide a food photographs with individual food portions during the interview, which was useful for more clear estimation. Based on data collected on FFQ, the daily total energy intake and other nutrients can be calculated according to Chinese Food Composition Table [18].

After excluding uncommon dietary items (with average intake < 2 serving/month), the 109 food items in the FFQ were grouped into 17 food groups based on the similarity of food type, nutrient profiles as well as local eating habits in local Chinese population (Supplementary Table S1). This approach has also been adopted by previous studies, and it could reduce the complexity of dietary data [6–8]. Based on the daily consumption of the 17 food groups that was not energy adjustment, factor analysis was used to derive dietary patterns. The food groups (factors) were rotated by orthogonal transformation (Varimax rotation function), resulting in uncorrelated, independent factors. Major factors retained were based on eigenvalue greater than 1.0, the scree plot (Supplementary Fig. S1), and factor interpretability, which were commonly used in breast cancer studies [19].

Factor loadings represent correlation coefficients between the food groups and the dietary pattern. The derived factors (patterns) were labeled as major food groups with higher factor loading as well as the interpretation of the data. This study identified two primary factors, which were labeled “grain and animal food pattern” and “vegetables and fruits pattern”. The factor score for each dietary pattern was calculated for each participant by summing intakes of food groups weighted by their factor loading [6]. Each individual was assigned a score for each identified dietary pattern, which reflect one’s conformity with that pattern; a higher factor score suggested better conformity with that pattern.

### QoL assessment using EORTC QLQ-C30

EORTC QLQ-C30 was used to measure patients’ QoL at the time of each assessment [20]. EORTC QLQ-C30 was designed to assess a range of cancer-specific QoL issues relevant to a broad spectrum of cancer patients [21]. This questionnaire consisted of 30 cancer-specific questions with multiple-point scales, including a global health status/QoL scale, five functional scales (physical, role, emotional, cognitive and social), nine symptom scales (fatigue, nausea and vomiting and pain, dyspnea, insomnia, appetite loss, constipation, diarrhea and financial difficulty). According to the EORTC QLQ-C30 Scoring Manual, multiple-point scales were transformed into standard scores (from 0 to 100) in the analysis. High scores on global health status/QoL and functioning scales represented good QoL, while high scores on the symptom scales indicated more severe symptoms [22].

### Statistical analysis

Based on data obtained at 18-month follow-up, patients were categorized into three groups (tertile 1, tertile 2 and tertile 3) according to the tertiles of factor scores in each dietary pattern and then the characteristics of participants were compared. Analysis of variance was used for continuous data and chi-square test for categorical data. Multivariable linear regression models were used to investigate the association between dietary pattern and QoL items cross-sectionally at 18-month follow-up. QoL items were log_10_ transformed to fulfill the normal distribution assumption, and the β values presented in the table were back-transformed by base 10 exponent to better interpret the results. The potential confounders were introduced in models using the enter method, which were identified based on the theoretical considerations, the previous literature, and the results of univariate analyses. Collinearity diagnostics were applied to exclude multicollinearity of variables. The following covariables were adjusted in the first model: age at follow-up (years; continuous), education level (high school or below, college or above), household income (< 15,000, 15,000–30,000, ≥ 30,000HKD/month), total number of comorbidities (0, 1, ≥ 2), menopausal status at follow-up (pre-menopausal, post-menopausal), AJCC stage (I, II, III, I–III without detail), ER status (positive, negative, missing), PR status (positive, negative, missing), current usage of adjuvant hormonal therapy (yes, no), BMI at follow-up (kg/m2; continuous), level of physical activity at follow-up (MET-hours/week; continuous), total and energy intake at follow-up (kcal/day; continuous). In the second model, factor scores of the other dietary pattern (when one of the two dietary patterns were being analyzed; continuous) were further adjusted. In addition, Generalized Estimating Equations (GEE) models were also used to analyze the longitudinal associations between each dietary pattern and QoL over four follow-ups [23]. The above-mentioned covariables and follow-up time-points (baseline, 18, 36 and 60 months; defined as 0.25 year, 1.5 year, 3 year and 5 year according to the median interval between the diagnosis of breast cancer and each follow-up in the GEE model) were adjusted in the multivariable analysis. All analyses were performed using SPSS 26.0, and *P* value < 0.05 based on two-sided analysis were considered statistically significant.

## Results

### Two major dietary patterns

Using post-diagnosis dietary intake assessed at 18-month follow-up, two major dietary patterns were identified by factor analysis. The representative food groups with their factor loadings in the dietary pattern are shown in Table 1. The first, termed “grain and animal food pattern”, was characterized by high factor loadings for refined grain, red meat, fish and seafood, oil, cakes and snacks, processed meat, eggs. The second, termed “vegetables and fruits pattern”, was characterized by high factor loadings for leaf vegetables, other vegetables, potato, fruits and legumes. These two factors explained 38.38% of the total variance (26.23% and 12.15%, respectively). Patients’ factor scores for each dietary pattern reflected one’s adherence to that dietary pattern. In this study, the median value (range) of factor scores for “grain and animal food pattern” and “vegetables and fruits pattern” were − 0.1 (− 3.4–5.1) and − 0.2 (− 2.1–5.3), respectively. Based on dietary intake assessed at baseline, 36- and 60-month follow-up, two similar dietary patterns were also observed (data not shown).

**Table 1:** Representative food groups^a^ for the two major dietary patterns identified by principal component analysis at 18-month follow-up

Foods/food groups	Factor 1 Grain and animal food pattern	Factor 2 Vegetable and fruit pattern
Refined grain	0.722	
Red meat	0.478	
Fish and seafood	0.411	
Oil	0.408	
Cakes and snacks	0.302	
Processed meat	0.266	
Eggs	0.262	
Leaf vegetables		0.932
Other vegetables		0.489
Fruits		0.390
Potato		0.314
Legumes		0.281
Variance explained (%)	26.228	12.154

^a^Representative food groups were selected based on factor loadings > 0.25

### Patients’ characteristics according to the tertiles of factor score in each dietary pattern at 18-month follow-up

Patients’ characteristics were compared according to the tertiles of factor score in each dietary pattern, and the results are summarized in Table 2. Patients in the highest tertile of “grain and animal food pattern” were younger, more likely to be pre-menopausal and had higher energy intake. In addition, compared to women in the lowest and highest tertile of “grain and animal food pattern”, the proportion of patients who were married was higher in the second tertiles. Patients in the highest tertile of “vegetables and fruits pattern” attained higher education level, were more likely to have normal BMI, were more physically active and had higher energy intake. Additionally, patients grouped into the second tertile factor score of “vegetables and fruits pattern” were more likely to be married, ER-positive, PR-positive and had higher household income.

**Table 2:** Characteristics of patients according to the tertiles of factor scores of each dietary pattern at 18-month follow-up

	Grain and animal food pattern				Vegetables and fruits pattern			
	Tertile 1	Tertile 2	Tertile 3	*P*	Tertile 1	Tertile 2	Tertile 3	*P*
Number of patients	408	410	408		409	409	408	
Factor score, median (range)	− 0.9 (-3.4 to -0.5)	− 0.1 (-0.5 to 0.3)	0.9 (0.3 to 5.1)		− 0.9 (-2.1 to − 0.5)	− 0.2 (− 0.5 to 0.3)	0.8 (0.3 to 5.3)	
Follow-up time since diagnosis, months, mean (SD)	19.1 (3.6)	19.2 (3.4)	19.5 (3.9)	0.219	19.3 (3.5)	19.1 (3.4)	19.6 (3.9)	0.135
Age at follow-up, years, mean (SD)	55.0 (9.2)	54.1 (8.8)	52.4 (8.9)	** < 0.0001**	54.4 (9.3)	53.5 (9.3)	53.6 (8.5)	0.260
Education level, %				0.269				0.018
High school or below	83.6	87.6	85.5		88.3	86.8	81.6	
Collage or above	16.4	12.4	14.5		11.7	13.2	18.4	
Marital status, %				**0.020**				**0.040**
Married or cohabitation	67.9	75.9	68.7		66.7	74.8	70.6	
Unmarried or divorced	32.1	24.1	32.3		33.3	25.5	29.4	
Household income (HKD/month), %				0.900				**0.008**
< 15,000	48.0	47.8	45.1		49.6	41.3	50.0	
15,000–30,000	30.4	31.5	33.1		32.8	31.8	30.4	
≥ 30,000	21.6	20.7	21.8		12.6	26.9	19.6	
BMI at follow-up, kg/m^2^, %				0.450				**0.007**
Underweight (< 18.5)	6.1	4.6	4.9		4.4	8.3	2.9	
Normal (18.5–22.9)	48.0	44.9	43.9		44.7	41.6	50.5	
Overweight (23–24.9)	20.3	22.4	19.4		20.5	20.5	21.1	
Obese (≥ 25)	23.5	28.0	31.9		30.3	29.6	25.5	
Number of comorbidities, %				0.056				0.094
None	57.8	60.5	64.7		57.9	62.3	62.7	
1	25.5	26.8	25.5		25.9	24.4	27.5	
≥ 2	16.7	12.7	9.8		16.1	13.3	9.8	
Parity, %				0.916				0.212
0	22.1	20.7	24.3		22.2	19.5	25.2	
1	22.8	22.7	23.0		22.0	25.2	21.3	
2	36.8	39.0	35.8		35.8	37.2	38.8	
≥ 3	18.4	17.6	16.9		20.0	18.1	14.7	
Menopause status at follow-up, %				**0.016**				0.609
Pre-menopausal	18.4	16.6	24.3		79.5	79.5	81.9	
Post-menopausal	81.6	83.4	75.7		20.5	20.5	18.1	
AJCC stage at diagnosis				0.056				0.533
I	33.1	30.2	31.4		30.1	32.5	32.1	
II	51.4	48.8	44.4		50.6	46.2	47.8	
III	15.5	20.3	24.0		18.6	21.3	19.4	
I–III without detail	0.5	0.7	0.2		0.7	0	0.7	
Histology, %				0.668				0.798
IDC	90.0	90.0	89.0		89.0	90.5	89.5	
ILC	2.2	3.7	3.4		3.9	2.7	2.7	
Others	7.8	6.3	7.6		7.1	6.8	7.8	
ER status, %				0.204				**0.036**
Positive	75.2	74.6	70.9		71.6	77.2	71.8	
Negative	23.3	22.9	25.2		24.2	21.8	25.5	
Missing	1.5	2.4	3.9		4.2	1.0	2.7	
PR status, %				0.397				**0.015**
Positive	56.6	59.1	53.7		55.7	57.9	55.6	
Negative	41.2	38.0	42.4		39.1	41.1	41.4	
Missing	2.2	2.9	3.9		5.1	1.0	2.9	
HER2 status, %				0.479				0.579
Positive	29.9	27.1	24.5		25.7	29.1	26.7	
Negative	66.9	70.0	71.6		70.4	67.2	70.8	
Missing	3.2	2.9	3.9		3.9	3.7	2.5	
Type of surgery				0.949				0.596
Mastectomy	65.4	64.4	64.7		66.7	64.3	63.4	
Conservation	34.6	35.6	35.3		33.3	35.7	36.5	
Chemotherapy, %				0.062				0.666
Yes	77.5	83.7	82.1		82.4	80.0	80.9	
No	22.5	16.3	17.9		17.6	20.0	19.1	
Radiation therapy, %				0.141				0.476
Yes	66.9	72.2	72.5		71.1	72.1	68.4	
No	33.1	27.8	27.5		28.9	27.9	31.6	
Hormone therapy, %				0.108				0.579
Yes	77.9	72.9	78.7		76.0	78.2	75.2	
No	22.1	27.1	21.3		24.0	21.8	24.8	
Physical activity level, MET-hours/week, mean (SD)	10.9 (12.8)	10.0 (13.6)	9.5 (13.6)	0.314	7.5 (11.4)	10.0 (12.2)	13.0 (15.5)	** < 0.0001**
Energy intake, kcal/day, mean (SD)	1083.7 (252.5)	1314.7 (251.2)	1686.8 (389.2)	** < 0.0001**	1155.1 (279.7)	1354.7 (355.1)	1575.7 (413.0)	** < 0.0001**

*SD* standard deviation, *HKD* Hong Kong dollars, *BMI* body mass index, *AJCC* American joint Committee on cancer, *IDC* invasive ductal carcinoma, *ILC* invasive lobular carcinoma, *DCIS* ductal carcinoma in situ, *ER* estrogen receptor, *PR* progesterone receptor, *HER 2* human epidermal-growth-factor receptor 2, *MET* metabolic equivalent of task

Bold face: *P* < 0.05

### Cross-sectional investigation of the association between post-diagnosis dietary pattern and QoL at 18-month follow-up

Multivariable linear regressions (Table 3) showed that after controlling for the potential confounders, “grain and animal food pattern” was inversely associated with fatigue and dyspnea. An increase of 1 point of “grain and animal food pattern” score was associated with a decrease of fatigue score by 2.238 (*P* = 0.010) and a decrease of dyspnea score by 1.986 (*P* = 0.024). In multivariable linear regression models (Table 4), higher vegetables and fruits food intake was positively associated with scores for global health status/QoL, but inversely associated with scores in loss of appetite and diarrhea. An increase of 1 point of “vegetables and fruits pattern” score was associated with an increase of global health status/QoL score by 2.195 (*P* = 0.002); while an increase of 1 point of “vegetables and fruits pattern” score was associated with a decrease of loss of appetite score by 1.058 (*P* = 0.040) and a decrease of diarrhea score by 1.342 (*P* = 0.009). Based on the evidence-based interpretation guidelines by Cocks et al. [24], the difference in individual QoL domains/symptoms were classified as trivial.

**Table 3:** Multivariate linear regression analyses of the factor scores of “grain and animal food pattern” on QoL in Chinese women with early-stage breast cancer at 18-month follow-up

EORTC QLQ-C30	Univariate analysis		Model 1		Model 2	
	*B* (95%CI)	*P*	*B* (95%CI)	*P*	*B* (95%CI)	*P*
Global health status/QoL	− 0.679 (− 1.664 to − 0.306)	0.262	− 0.786 (− 2.255 to 0.682)	0.551	0.825 (− 0.935 to 2.586)	0.226
Functioning						
Physical functioning	− 0.064 (− 0.696 to 0.568)	0.831	0.343 (− 0.602 to 1.287)	0.555	0.886 (− 0.249 to 2.022)	0.176
Role functioning	− 1.531 (− 2.349 to − 0.712)	0.012	− 0.676 (− 1.911 to − 0.559)	0.326	− 0.610 (− 2.097 to 0.876)	0.682
Emotional functioning	− 0.950 (− 2.001 to 0.100)	0.466	0.078 (− 1.493 to 1.648)	0.978	1.099 (− 0.788 to 2.987)	0.472
Cognitive functioning	0.031 (− 1.037 to 1.099)	0.822	1.168 (− 0.445 to 2.781)	0.180	1.618 (− 0.323 to 3.559)	0.104
Social functioning	− 0.696 (− 1.570 to 0.178)	0.141	0.385 (− 0.933 to 1.703)	0.855	0.473 (− 1.113 to 2.060)	0.745
Symptoms						
Fatigue	0.443 (− 0.664 to 1.550)	0.440	− 1.775 (− 3.431 to − 0.119)	0.044	− 2.238 (− 4.230 to − 0.245)	**0.010**
Nausea and vomiting	0.227 (− 1.138 to 0.591)	0.057	0.048 (− 0.499 to 0.594)	0.654	− 0.186 (− 0.844 to 0.472)	0.974
Pain	0.916 (− 3.373 to 2.204)	0.163	− 0.877 (− 2.792 to 1.039)	0.444	− 1.209 (− 3.515 to 1.097)	0.261
Dyspnea	0.138 (− 0.812 to 1.087)	0.608	− 1.438 (− 2.862 to − 0.013)	0.048	− 1.986 (− 3.700 to − 0.272)	**0.024**
Insomnia	− 0.493 (− 2.100 to 1.114)	0.643	− 1.029 (− 3.467 to 1.049)	0.803	− 1.068 (− 4.002 to 1.866)	0.540
Loss of appetite	0.043 (− 0.691 to 0.776)	0.783	− 0.378 (− 1.486 to 0.729)	0.847	− 1.155 (− 2.486 to 0.176)	0.193
Constipation	0.043 (− 1.061 to 1.148)	0.546	0.544 (− 1.130 to 2.218)	0.347	− 0.296 (− 2.309 to 1.717)	0.899
Diarrhea	0.508 (− 0.193 to 1.209)	0.084	0.696 (− 0.349 to 1.742)	0.144	− 0.289 (− 1.543 to 0.965)	0.810
Financial impact	0.799 (− 0.530 to 2.128)	0.054	− 1.632 (− 3.598 to 0.334)	0.192	− 1.074 (− 3.440 to 1.292)	0.281

Multivariate linear regressions were used to analyze the association of “grain and animal food pattern” and QoL. QoL items were log_10_ transformed in multivariate linear regressions, and the β values (95%CI) presented in the table were back-transformed by base 10 exponent to better interpret the results. The potential confounders were introduced in the models using the enter method. Model 1, adjusted for age at 18-month follow-up, education level, income status, total number of comorbidities, menopausal status at 18-month follow-up, AJCC stage, ER status and PR status, current usage of adjuvant hormonal therapy, BMI at 18-month follow-up, level of physical activity, total energy intake; Model 2, further adjusted for factor scores of “vegetables and fruits pattern”

*B* Unstandardized coefficient of linear regression model, *95%CI* 95% confidence interval, *QoL* quality of life, *EORTC QLQ-C30* European Organization for Research and Treatment of Cancer Quality of Life Questionnaire Core 30, *AJCC* American joint Committee on cancer, *ER* estrogen receptor, *PR* progesterone receptor

Bold face: *P* < 0.05

**Table 4:** Multivariate linear regression analyses of the factor score of “vegetables and fruits pattern” on QoL in Chinese women with early-stage breast cancer at 18-month follow-up

EORTC QLQ-C30	Univariate analysis		Model 1		Model 2	
	*B* (95%CI)	*P*	*B* (95%CI)	*P*	*B* (95%CI)	*P*
Global health status/QoL	1.693 (− 0.712 to 2.674)	0.003	1.847 (0.739 to 2.956)	0.004	2.195 (0.862 to 3.529)	**0.002**
Functioning						
Physical functioning	0.487 (− 0.144 to 1.118)	0.132	0.367 (− 0.348 to 1.082)	0.337	0.741 (− 0.120 to 1.601)	0.121
Role functioning	− 0.259 (− 1.082 to 0.564)	0.790	0.346 (− 0.589 to 1.282)	0.249	0.089 (− 1.037 to 1.215)	0.465
Emotional functioning	0.411 (− 0.640 to 1.462)	0.363	0.928 (− 0.261 to 2.116)	0.305	1.391 (− 0.039 to 2.821)	0.210
Cognitive functioning	− 0.074 (− 1.142 to 0.993)	0.865	− 0.069 (− 1.291 to 1.154)	0.983	0.613 (− 0.857 to 2.083)	0.357
Social functioning	− 0.457 (− 1.332 to 0.417)	0.327	− 0.079 (− 1.078 to 0.919)	0.873	0.120 (− 1.082 to 1.322)	0.754
Symptoms						
Fatigue	0.453 (− 0.653 to 1.560)	0.873	0.313 (− 0.944 to 1.569)	0.815	− 0.630 (− 2.139 to 0.879)	0.102
Nausea and vomiting	− 0.228 (− 0.592 to 0.137)	0.517	− 0.240 (− 0.654 to 0.174)	0.449	− 0.318 (− 0.816 to 0.180)	0.542
Pain	0.321 (− 0.968 to 1.610)	0.892	0.056 (− 1.395 to 1.508)	0.763	− 0.453 (− 2.200 to 1.293)	0.381
Dyspnea	0.188 (− 0.761 to 1.138)	0.683	0.090 (− 0.991 to 1.171)	0.959	− 0.747 (− 2.046 to 0.552)	0.224
Insomnia	0.121 (− 1.486 to 1.728)	0.699	0.397 (− 1.450 to 2.244)	0.641	− 0.053 (− 2.276 to 2.170)	0.467
Loss of appetite	− 0.570 (− 1.303 to 0.162)	0.068	− 0.571 (− 1.410 to 0.268)	0.110	− 1.058 (− 2.066 to − 0.049)	**0.040**
Constipation	− 1.088 (− 2.191 to 0.015)	0.054	− 1.020 (− 2.286 to 0.247)	0.060	− 1.144 (− 2.669 to 0.381)	0.102
Diarrhea	− 1.090 (− 1.788 to − 0.391)	0.004	− 1.220 (− 2.010 to − 0.431)	0.003	− 1.342 (− 2.292 to − 0.392)	**0.009**
Financial impact	1.636 (0.309 to 2.963)	0.031	1.212 (− 0.277 to 2.702)	0.463	0.760 (− 1.032 to 2.552)	0.992

Multivariate linear regressions were used to analyze the association of “vegetables and fruits pattern” and QoL. QoL items were log_10_ transformed in multivariate linear regressions, and the β values presented in the table were back-transformed to better interpret the results. The potential confounders were introduced in the models using the enter method. Model 1, adjusted for age at 18-month follow-up, education level, income status, total number of comorbidities, menopausal status at 18-month follow-up, AJCC stage, ER status and PR status, current usage of adjuvant hormonal therapy, BMI at 18-month follow-up, level of physical activity, total energy intake; Model 2, further adjusted for factor scores of “grain and animal food pattern”

*B* Unstandardized coefficient of linear regression model, *95%CI* 95% confidence interval, *QoL* quality of life, *EORTC QLQ-C30* European Organization for Research and Treatment of Cancer Quality of Life Questionnaire Core 30, *AJCC* American joint Committee on cancer, *ER* estrogen receptor, *PR* progesterone receptor

Bold face: *P* < 0.05

### Longitudinal investigation of the association between each dietary pattern and QoL over four follow-ups

Based on the GEE analysis, after controlling for four follow-up time-points and other covariables, each dietary pattern was significantly associated with several items of QoL. An increase of 1 point of “grain and animal food pattern” score was associated with a deterioration of role functioning score by 0.744 (Table 5, *P* = 0.045), an improvement of dyspnea score by 0.092 (*P* < 0.001) and an improvement of constipation score by 1.355 (*P* = 0.001). An increase of 1 point of “vegetables and fruits pattern” score was associated with increases of global health status/QoL score by 1.282 (Table 6, *P* = 0.001), physical functioning score by 0.545 (*P* = 0.035), emotional functioning score by 1.426 (*P* < 0.001) and cognitive functioning by 0.822 (*P* = 0.048); this was also associated with decreases of nausea and vomiting score by 0.382 (*P* = 0.016), dyspnea score by 0.570 (*P* < 0.001), insomnia score by 1.412 (*P* = 0.025), loss of appetite score by 0.722 (*P* = 0.016), constipation score by 2.028 (*P* < 0.001) and diarrhea score by 0.929 (*P* = 0.001). According to the evidence-based interpretation guidelines by Cocks et al. [25], all differences in individual QoL domains/symptoms were classified as trivial.

**Table 5:** GEE models to analyze the factor scores of “grain and animal food pattern” on QoL in Chinese women with early-stage breast cancer over four follow-ups

EORTC QLQ-C30	Univariate analysis		Model 1		Model 2	
	*B* (95%CI)	*P*	*B* (95%CI)	*P*	*B* (95%CI)	*P*
Global health status/QoL	− 0.691 (− 1.323 to − 0.058)	0.032	− 0.349 (− 1.112 to 0.413)	0.370	0.114 (− 0.665 to 0.892)	0.775
Functioning						
Physical functioning	− 0.612 (− 1.021 to − 0.204)	0.003	− 0.319 (− 0.838 to 0.200)	0.228	− 0.122 (− 0.676 to 0.431)	0.665
Role functioning	− 1.293 (− 1.852 to − 0.734)	< 0.001	− 0.891 (− 1.583 to − 0.199)	0.012	− 0.744 (− 0.147 to − 0.017)	**0.045**
Emotional functioning	− 1.323 (− 1.978 to − 0.667)	< 0.001	0.020 (0.020 to 0.020)	< 0.001	0.535 (1.382 to 1.528)	0.216
Cognitive functioning	− 0.602 (− 1.237 to − 0.033)	0.063	0.423 (0.423 to 0.423)	< 0.001	0.720 (− 0.144 to 1.583)	0.102
Social functioning	− 1.111 (− 1.685 to − 0.536)	< 0.001	− 0.284 (− 1.001 to 0.432)	0.436	− 0.049 (− 0.829 to 0.731)	0.902
Symptoms						
Fatigue	1.141 (0.435 to 1.847)	0.002	− 0.199 (− 1.085 to 0.688)	0.661	− 0.491 (− 1.439 to 0.457)	0.311
Nausea and vomiting	0.230 (− 0.039 to 0.500)	0.094	− 0.050 (− 0.332 to 0.123)	0.726	− 0.188 (− 0.513 to 0.137)	0.256
Pain	1.486 (0.737 to 2.236)	< 0.001	0.543 (− 0.415 to 1.501)	0.267	0.534 (− 0.468 to 1.536)	0.296
Dyspnea	1.033 (0.432 to 1.634)	< 0.001	0.113 (0.113 to 0.113)	< 0.001	− 0.092 (− 0.092 to − 0.092)	** < 0.001**
Insomnia	− 0.147 (− 1.096 to 0.802)	0.762	− 0.653 (− 1.821 to 0.514)	0.273	− 1.163 (− 2.373 to 0.047)	0.060
Loss of appetite	0.319 (− 0.158 to 0.796)	0.190	0.279 (− 0.294 to 0.852)	0.340	0.019 (− 0.617 to 0.654)	0.954
Constipation	0.241 (− 0.379 to 0.851)	0.447	− 0.623 (− 0.623 to − 0.623)	< 0.001	− 1.355 (− 2.174 to − 0.536)	**0.001**
Diarrhea	0.815 (0.384 to 1.246)	< 0.001	0.400 (− 0.112 to 0.911)	0.126	0.065 (− 0.498 to 0.627)	0.822
Financial impact	1.122 (0.301 to 1.944)	0.007	− 0.392 (− 1.266 to 0.481)	0.379	− 0.456 (− 1.365 to 0.453)	0.325

GEE models were used to analyze the association of “grain and animal food pattern” and QoL longitudinally over four follow-ups. The potential confounders were introduced in the models using the enter method. Model 1, adjusted for age at follow-up, education level, income status, total number of comorbidities, menopausal status at follow-up, AJCC stage, ER status and PR status, current usage of adjuvant hormonal therapy, BMI at follow-up, level of physical activity at follow-up, total energy intake at follow-up; Model 2, further adjusted for factor scores of “vegetables and fruits pattern”

*B* Unstandardized coefficient of GEE model, *95%CI* 95% confidence interval, *QoL* quality of life, *EORTC QLQ-C30* European Organization for Research and Treatment of Cancer Quality of Life Questionnaire Core 30, *AJCC* American joint Committee on cancer, *ER* estrogen receptor, *PR* progesterone receptor

Bold face: *P* < 0.05

**Table 6:** GEE models to analyze the factor scores of “vegetables and fruits pattern” on QoL in Chinese women with early-stage breast cancer over four follow-ups

EORTC QLQ-C30	Univariate analysis		Model 1		Model 2	
	*B* (95%CI)	*P*	*B* (95%CI)	*P*	*B* (95%CI)	*P*
Global health status/QoL	1.104 (0.400 to 1.807)	0.002	1.251 (0.535 to 1.967)	0.001	1.282 (0.545 to 2.019)	**0.001**
Functioning						
Physical functioning	0.493 (0.033 to 0.953)	0.036	0.578 (0.101 to 1.056)	0.018	0.545 (0.037 to 1.053)	**0.035**
Role functioning	0.163 (− 0.465 to 0.732)	0.610	0.609 (− 0.065 to 1.282)	0.077	0.406 (− 0.301 to 1.114)	0.260
Emotional functioning	0.542 (− 0.159 to 1.243)	0.130	1.282 (0.539 to 2.023)	0.001	1.426 (0.653 to 2.200)	** < 0.001**
Cognitive functioning	0.110 (− 0.611 to 0.831)	0.765	0.626 (− 0.129 to 1.382)	0.104	0.822 (0.007 to 1.637)	**0.048**
Social functioning	0.172 (− 0.457 to 0.801)	0.591	0.666 (0.013 to 1.318)	0.045	0.652 (− 0.054 to 1.358)	0.070
Symptoms						
Fatigue	− 0.181 (− 0.929 to 0.567)	0.635	− 0.676 (− 1.454 to 0.102)	0.088	− 0.810 (− 1.637 to 0.018)	0.055
Nausea and vomiting	− 0.255 (− 0.507 to − 0.003)	0.047	− 0.331 (− 0.605 to − 0.057)	0.018	− 0.382 (− 0.694 to − 0.071)	**0.016**
Pain	0.382 (− 0.414 to 1.178)	0.347	− 0.169 (− 0.998 to 0.661)	0.690	− 0.024 (− 0.892 to 0.845)	0.957
Dyspnea	− 0.043 (− 0.718 to 0.633)	0.902	− 0.545 (− 0.545 to − 0.545)	< 0.001	− 0.570 (− 0.570 to − 0.570)	** < 0.001**
Insomnia	− 0.672 (− 1.781 to 0.437)	0.235	− 1.096 (− 2.287 to 0.095)	0.071	− 1.412 (− 2.647 to − 0.177)	**0.025**
Loss of appetite	− 0.668 (− 1.188 to − 0.148)	0.012	− 0.727 (− 1.264 to − 0.189)	0.008	− 0.722 (− 1.311 to − 0.132)	**0.016**
Constipation	− 1.103 (− 1.797 to − 0.409)	0.002	− 1.659 (− 2.372 to 0.947)	< 0.001	− 2.028 (− 2.775 to − 1.281)	** < 0.001**
Diarrhea	− 0.590 (− 1.031 to − 0.148)	0.009	− 0.946 (− 1.450 to − 0.442)	< 0.001	− 0.929 (− 1.481 to − 0.377)	**0.001**
Financial impact	0.625 (− 0.278 to 0.175)	0.175	− 0.052 (− 0.939 to 0.834)	0.908	− 0.176 (− 1.102 to 0.749)	0.709

GEE models were used to analyze the association of “vegetables and fruits pattern” and QoL longitudinally over four follow-ups. The potential confounders were introduced in the models using the enter method. Model 1, adjusted for age at follow-up, education level, income status, total number of comorbidities, menopausal status at follow-up, AJCC stage, ER status and PR status, current usage of adjuvant hormonal therapy, BMI at follow-up, level of physical activity at follow-up, total energy intake at follow-up; Model 2, further adjusted for factor scores of “grain and animal food pattern”

*B* Unstandardized coefficient of GEE model, *95%CI* 95% confidence interval, *QoL* quality of life, *EORTC QLQ-C30* European Organization for Research and Treatment of Cancer Quality of Life Questionnaire Core 30, *AJCC* American joint Committee on cancer, *ER* estrogen receptor, *PR* progesterone receptor

Bold face: *P* < 0.05

## Discussion

Using data assessed at baseline, 18-, 36- and 60-month follow-up from a prospective cohort of Chinese women with breast cancer, two similar dietary patterns were identified at each follow-up time-points, namely “grain and animal food pattern” and “vegetables and fruits pattern”. Longitudinal analysis showed that higher adherence to each dietary pattern was significantly associated with QoL. Higher intake of “grain and animal food pattern” was associated with worse role functioning, while less symptoms in dyspnea and constipation. Women with a higher consumption of vegetables and fruits tended to have better scores for global health status/QoL, physical functioning, emotional functioning and cognitive functioning, as well as less symptoms in nausea and vomiting, dyspnea, insomnia, loss of appetite, constipation and diarrhea.

The relationship between dietary pattern and QoL has only been investigated in a cross-sectional study among 232 Korean breast cancer survivors. This study used 3-day dietary record to assess dietary intake, in which two major dietary patterns were identified, “healthy dietary pattern” and “western dietary pattern” [26]. Higher “healthy dietary pattern” score (as assessed by EORTC QLQ-C30) was associated with milder dyspnea but more severe symptom of insomnia. Of note, even though the 3-day dietary record was regarded as a gold standard to validate FFQ, they cannot comprehensively represent the habitual dietary intake over a relative long time. The dietary patterns identified and their associations with QoL were different with the present results, which may reflect the different dietary habits in Hong Kong and Korea. A few studies have evaluated the impact of quality of diet on QoL in breast cancer survivors. In the Health, Eating, Activity and Lifestyle (HEAL) study, dietary intake was assessed by FFQ, and those dietary data was used to evaluate diet quality by the Diet Quality Index (DQI) [27]. The DQI assessed an individual’s adherence to eight dietary recommendations from the National Academy of Sciences regarding fat, saturated fat, cholesterol, sodium, fruits/vegetables, grain, protein and calcium intake. According to the level of compliance, the diet quality scores can be categorized into four groups: excellent, good, fair and poor. Compared to women with poor diet quality, those with excellent diet quality had significantly higher Mental component summary (MCS) scores as evaluated by Medical Outcomes Study Short Form Survey 36 (SF-36) [27]. In another report from the cohort of the HEAL study, diet quality was evaluated by the Healthy Eating Index 2010 (HEI-2010) [28]. Based on their dietary data, a HEI-2010 score ranging from 0 to 100 points was calculated for each patient. According to the HEI-2010 score, patients were classified into quartiles (Q4–Q1, from better-quality to poor-quality). The results showed that higher quality of diet was associated with reduced cancer-related fatigue [28]. Mosher et al. reported the association between diet and QoL among elderly cancer survivors, including those with breast cancer [29]. Overall diet quality was assessed on two days based on 24-h recalls, and subsequently evaluated by HEI-2005 (HEI-2005). Each participant had a HEI-2005 score, ranging from 0 (worst) to 100 (best). Diet quality was positively related to Physical component summary (PCS) score as measured by SF-36 [29].

The present study found that patients with higher intake of grain and animal food were younger and more likely to be pre-menopausal. These findings contributed to our understanding of breast cancer patients’ dietary habits, whereby healthcare professionals should pay more attention to educate younger patients to limit the consumption of red meat and processed meat. In addition, patients with higher intake of vegetables and fruits had higher education level, were more likely to have normal BMI and be more physically active. It reflected that patients who had higher education level were more likely to adopt a healthier dietary habit. Furthermore, patients who cared about their diet also tend to adopt a healthier lifestyle habit, being more physically active and more likely to maintain a normal weight.

The findings in cross-sectional analysis suggested that higher adherence to “grain and animal food pattern” at post-diagnosis may be inversely related to fatigue and dyspnea. These associations have not been reported in previous studies and the potential mechanisms underlying such associations were not clear. Of note, patients with higher intake of grain and animal food were younger; and younger patients have reported less fatigue [30] and dyspnea [31] than older ones. Hence, these associations may be influenced by patients’ age, although age at 18-month follow-up has been adjusted in the multivariable analyses. Findings from longitudinal analysis showed similar associations between “grain and animal food pattern” and dyspnea. It also indicated that “grain and animal food pattern” was inversely associated with role functioning. Role functioning was a core construct of QoL, which reflects patients’ ability to fulfill occupational and social roles in their survivorship [32]. This association has not been reported in previous studies and the potential mechanism underlying such association was not clear. There is, however, some evidence, which showed that western dietary pattern (characterized as refined grain, red meat, processed meat, and food with high-fat, similar to the “grain and animal food pattern” in the present study) was associated with higher level of low-grade inflammation and might induce subsequent cognitive decline [33, 34]. The association between “grain and animal food pattern” and constipation in the longitudinal analysis has not been reported yet. The potential mechanism mediating such association is unknown and warrants further studies to explore.

The cross-sectional analysis suggested higher adherence to “vegetables and fruits pattern” was associated with better global health status/QoL and less symptoms of loss of appetite and diarrhea. Longitudinal analysis further supported those associations, and also indicated that high intake of “vegetables and fruits pattern” was associated with better physical functioning, emotional functioning and cognitive functioning, as well as less symptoms in nausea and vomiting, dyspnea, insomnia and constipation. Such associations were consistent with a previous cross-sectional study in Chinese women with breast cancer, which included 3,344 women with most patients (63%) having survived for at least 5 years [3]. Participants were asked to report the frequency of vegetables and fruits intake in the past month and they underwent QoL assessment at the same time. EORTC QLQ-C30 was used to measure QoL. Compared to women who had vegetables ≤ 250 g/day, breast cancer survivors who consumed vegetables > 250 g/day had significantly higher scores in most QoL dimensions, including global health status/QoL, physical, cognitive and emotional functioning, as well as milder symptoms of dyspnea, insomnia, loss of appetite and constipation. In addition, compared to women who did not eat fruits every day, those who had fruits every day reported significantly higher scores in all QoL dimensions, except for symptom items [3]. The association between fruits and vegetables intake and QoL among cancer survivors has also been investigated in the US women [4]. Patients were defined as meeting the ACS 5-A-Day recommendation if they consumed at least 5 servings of fruits and vegetables per day. Results showed that breast cancer survivors who met the 5-A-Day recommendation had slightly yet significantly higher overall global health composite score compared with those who did not [4]. The potential mechanism underlying the association between specific dietary pattern or food item with QoL has not been fully elucidated. One mechanism may underlie the association between dietary intake and gastrointestinal symptoms: vegetables and fruits contain high quantities of dietary fiber which might improve gastrointestinal function, and relief constipation and diarrhea [35].

Based on data from a prospective cohort study with large sample size, this was the first study to investigate the associations between dietary patterns and QoL among Chinese breast cancer survivors. In addition, this study also used GEE models to control for follow-up time-points, which provided an opportunity to explore the relationship between dietary pattern and QoL in a longitudinal manner. Moreover, this study used a validated FFQ to measure dietary intake and collected patients’ dietary intake information over the previous year in a relatively accurate manner. One of the major limitations of the present study was that as in other dietary pattern studies, factor analysis used to identify dietary patterns involves some arbitrary decisions, including grouping of food items, method of factor rotation and labeling of dietary pattern [36]. In addition, the current study is of exploratory rather than confirmatory nature. As such, the positive effects of a vegetable-based diet on QoL outcomes in breast cancer patients reported in the present study should be further supported by confirmatory analyses with preregistered hypothesis and analysis protocols in future research. Furthermore, we have only adjusted the number of other comorbidities and could not consider of medication usages during each follow-up in the multivariate models due to lack of such data, and this might influence patients’ QoL.

Of note, cultural background of the dietary patterns should be considered in the interpretation of the results. For instance, higher intake of “vegetables and fruit pattern” tends to be a part of traditional Chinese diet, which is characterized by well-balanced dietary intake, high in fiber and low in saturated fats [37]. There may also be a possibility that the diet itself was not entirely accounting for the observed effects, but the associated whole lifestyle habits may be responsible. Patients who were conscious of a healthier dietary intake may also be more likely to adopt a healthy lifestyle habit. Although a wide range of covariates, including BMI, level of physical activity and energy intake, have been carefully adjusted in the analyses, potential residual confounding effects cannot be completely ruled out.

## Conclusions

The present study indicated that each dietary pattern was associated with several aspects of QoL. Higher intake of grain and animal foods was related to worse role functioning, while less symptoms in dyspnea and constipation. In addition, higher consumptions of vegetables and fruits were associated with better global health status/QoL, physical functioning, emotional functioning and cognitive functioning, as well as less severe symptom in nausea and vomiting, dyspnea, insomnia, loss of appetite, constipation and diarrhea. Although all the differences in individual QoL domains/symptoms were classified as trivial based on the evidence-based interpretation guidelines [24, 25], these findings hint to a potential beneficial effect on QoL for breast cancer patients following a healthy lifestyle habit. Such mild findings may be explained by potential reasons, such as the limited room for improvement at baseline QoL status, the exclusion of patients with other cancers or recurrence during follow-up, or the adoption of different approaches for pattern analysis. Furthermore, prospective follow-up data could confirm whether specific dietary pattern has impact on outcomes of breast cancer patients.

## Supplementary Information

Below is the link to the electronic supplementary material.

Supplementary file1 (DOCX 29 KB)

## Data Availability

All analyzed data during the current study were presented in the main manuscript and supplementary file. The original datasets are available from the corresponding author on reasonable request.
