# Supplementary material for: Association of high adherence to vegetables and fruits dietary pattern with quality of life among Chinese women with early-stage breast cancer
Source: Qual Life Res. 2021 Sep 16;31(5):1371–84. doi: 10.1007/s11136-021-02985-0 (PMC9023405; doi:10.1007/s11136-021-02985-0)
Supplement: Supplementary file 1 — Supplementary file1 (DOCX 29 KB) [file 11136_2021_2985_MOESM1_ESM.docx]

Supplementary materials


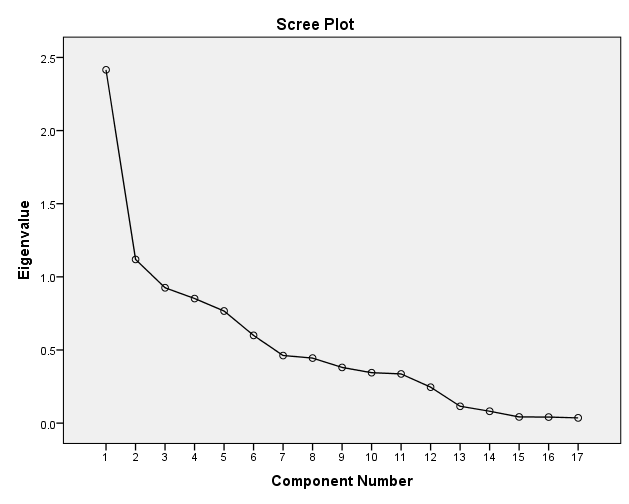


Supplementary Figure 1. The scree plot.

Supplementary Table 1. Grouping food items into food groups

| Food group | Food item |
| --- | --- |
| 1. Whole grain | Oatmeal, whole wheat bread, wheat gluten |
| 2. Refined grain | Rice, congee, noodles, pasta, plain roll, roll with filling, rice roll |
| 3. Cakes | Pancakes or waffles, cracker, cake, biscuits |
| 4. Poultry | Chicken with or without skin, duck, goose, pigeon, quail |
| 5. Red meat | Beef, pork, lamb, meat ball, liver |
| 6. Processed meat | Processed meat (eg. Bacon, sausages, luncheon meat, ham), Chinese sausage |
| 7. Fish and seafood | Freshwater fish, seawater fish, small fish with edible bone, shrimp, fish ball, seafood ball |
| 8. Eggs | Eggs or preserved eggs |
| 9. Dairy | Whole milk, whole milk powder, skim or low-fat milk, skim or low-fat milk powder, cream, ice cream, cheese, condensed milk, milkshake, milk tea, yogurt |
| 10. Leaf vegetables | Dark green leafy vegetable (e.g. including broccoli, Chinese cabbage, ChoiSum, kale, watercress, mustard, mustard, culiflower) |
| 11. Other vegetables | melons, tomato, eggplant, squash, cucumbers, radish and pepper, carrot, fresh corn, allium, fresh beans, mushrooms, fungi |
| 12. Potato | Potato, sweet potato |
| 13. Legumes | Pea, other beans |
| 14. Fruits | Citrus fruits (e.g. oranges, tangerines, grapefruit, kiwi), grapes, bananas, melons (e.g. watermelon, cantaloupe, honeydew), non-citrus fruits (e.g. apple, pear, apricot, peach, plum, mango, pineapple) |
| 15. Soy foods | Firm tofu (with or out brand name), soft tofu, wrapped tofu, bean curd puff, deep-fried bean curd, deep-fried preserved chou tofu, preserved hot bean curd, preserved bean curd with sesame oil, dried tofu, chauchow dried tofu, soybean, miso paste, green soybean, soybean sprout, layered tofu sheet, commercially prepared miso soup, bean curd sheet, deep-fried tofu stick, tofu stick, bean curd skin, vegetarian ham chicken, vegetarian duck, bean curd pudding, homemade or commercial no brand soymilk, commercially prepared brand soymilk, low sugar low fat brand soymilk, soymilk powder |
| 16. Nuts | Peanuts, walnut, almond, cashew nut, pistachio nut, pumpkin seed, sunflower seed |
| 17. Oil and fat | Cooking oil (e.g. com oil, peanut oil, olive oil，safflower oil), mayonnaise, butter, margarine |
